# Myxomavirus Anti-Inflammatory Chemokine Binding Protein Reduces the Increased Plaque Growth Induced by Chronic *Porphyromonas gingivalis* Oral Infection after Balloon Angioplasty Aortic Injury in Mice

**DOI:** 10.1371/journal.pone.0111353

**Published:** 2014-10-29

**Authors:** Alexandra R. Lucas, Raj K. Verma, Erbin Dai, Liying Liu, Hao Chen, Sheela Kesavalu, Mercedes Rivera, Irina Velsko, Sriram Ambadapadi, Sasanka Chukkapalli, Lakshmyya Kesavalu

**Affiliations:** 1 Division of Cardiovascular Medicine, Departments of Medicine and Molecular Genetics & Microbiology, College of Medicine, University of Florida, Gainesville, Florida, United States of America; 2 Department of Periodontology, College of Dentistry, University of Florida, Gainesville, Florida, United States of America; 3 Department of Oral Biology, College of Dentistry, University of Florida, Gainesville, Florida, United States of America; Boston University, United States of America

## Abstract

Thrombotic occlusion of inflammatory plaque in coronary arteries causes myocardial infarction. Treatment with emergent balloon angioplasty (BA) and stent implant improves survival, but restenosis (regrowth) can occur. Periodontal bacteremia is closely associated with inflammation and native arterial atherosclerosis, with potential to increase restenosis. Two virus-derived anti-inflammatory proteins, M-T7 and Serp-1, reduce inflammation and plaque growth after BA and transplant in animal models through separate pathways. M-T7 is a broad spectrum C, CC and CXC chemokine-binding protein. Serp-1 is a *ser*ine *p*rotease *in*hibitor (*serpin*) inhibiting thrombotic and thrombolytic pathways. Serp-1 also reduces arterial inflammation and improves survival in a mouse herpes virus (MHV68) model of lethal vasculitis. In addition, Serp-1 demonstrated safety and efficacy in patients with unstable coronary disease and stent implant, reducing markers of myocardial damage. We investigate here the effects of *Porphyromonas gingivalis*, a periodontal pathogen, on restenosis after BA and the effects of blocking chemokine and protease pathways with M-T7 and Serp-1. ApoE^−/−^ mice had aortic BA and oral *P. gingivalis* infection. Arterial plaque growth was examined at 24 weeks with and without anti-inflammatory protein treatment. Dental plaques from mice infected with *P. gingivalis* tested positive for infection. Neither Serp-1 nor M-T7 treatment reduced infection, but IgG antibody levels in mice treated with Serp-1 and M-T7 were reduced. *P. gingivalis* significantly increased monocyte invasion and arterial plaque growth after BA (P<0.025). Monocyte invasion and plaque growth were blocked by M-T7 treatment (P<0.023), whereas Serp-1 produced only a trend toward reductions. Both proteins modified expression of TLR4 and MyD88. In conclusion, aortic plaque growth in ApoE^−/−^ mice increased after angioplasty in mice with chronic oral *P. gingivalis* infection. Blockade of chemokines, but not serine proteases significantly reduced arterial plaque growth, suggesting a central role for chemokine-mediated inflammation after BA in *P. gingivalis* infected mice.

## Introduction

Atherosclerotic plaque growth is accelerated by hyperlipidemia, hypertension, and diabetes which cause arterial injury. Percutaneous intervention (PCI) with either balloon angioplasty (BA) or stent implant, is associated with a rapid recurrent plaque growth, termed restenosis, that is characterized by endothelial cell dysfunction, smooth muscle cell migration into the intima, and inflammatory macrophage and T cell activation [1], [2]. While acute thrombosis at sites of angioplasty and stent implant is well controlled with anti-platelet agents such as aspirin and clopidogrel, the causes for restenosis are only partially understood [1]–[3]. Prevention of restenosis is limited to the use of bare metal stents, which reduce restenosis from 30–50% after BA alone to 20–30%, and drug eluting stents which further reduce restenosis to 3–10%. Inflammatory macrophage and T cell invasion can drive both early and late unstable atherosclerotic plaque progression, and can also induce restenosis. While restenosis is considered a specialized form of rapid arterial plaque growth, it is, by definition, formed at sites of already developed atheroma and thus is influenced both by angioplasty injury and the underlying atherosclerotic plaque.

Periodontal disease (PD) is a multispecies, subgingival, biofilm-mediated disease and an estimated 5–20% of the world’s population suffer from chronic periodontitis [4]. Periodontitis is also believed to contribute to systemic diseases, including atherosclerotic vascular disease, diabetes mellitus, rheumatoid arthritis, and Alzheimer’s disease [5]–[7]. *P. gingivalis,* the most common oral pathogen, is reported to increase plaque growth after wire-induced femoral arterial injury in mice upon systemic infection with subcutaneous bacterial inoculations [8]. *Streptococcus mutans* similarly increases plaque after BA [9]. Prior studies with oral *P. gingivalis* infection in ApoE^−/−^ mice have demonstrated both periodontal disease and atherosclerosis [8], [10], [11] and genomic DNA from *P. gingivalis* has been detected in atherosclerotic plaque [12]. Apolipoprotein E (ApoE) is a ligand for receptors that clear remnants of chylomicrons and very low density lipoproteins. Lack of ApoE is, therefore, expected to cause accumulation in plasma of cholesterol-rich remnants whose prolonged circulation should be atherogenic. Apo E-deficient mice generated by gene targeting were used as a model to test this hypothesis and are known to for developing spontaneous atherosclerosis that is increased with balloon angioplasty [13], [14]. Macrophage and T cell invasion, as well as expression of Toll-like receptors (TLRs) 2 and 4, pro-inflammatory cytokines interleukin-6 (IL-6) and vascular cell adhesion molecule-1 (VCAM-1) were also detected after *P. gingivalis* infection [8], [15]–[18].

Viruses have developed potent anti-inflammatory proteins over millions of years of evolution that protect them from host immune defenses [19]–[26]. M-T7 and Serp-1 proteins increase viral pathogenesis in myxomaviral infection in European rabbits at picomolar concentrations, by blocking select steps in host inflammatory responses. M-T7 binds and inhibits C, CC, and CXC class chemokines through interfering with chemokine: glycosamnoglycan (GAG) interactions [19], [20] and Serp-1 is a *ser*ine *p*rotease *in*hibitor (*serpin*) that blocks tissue- and urokinase-type plasminogen activators (tPA, uPA, respectively), plasmin, and factor Xa (fXa) [21]–[24]. Infusion of purified M-T7 or Serp-1 proteins markedly reduces inflammatory cell invasion and arterial plaque in animal models of atherosclerosis, restenosis, and transplant [19]–[24]. Serp-1 has also been tested in a clinical trial in patients with unstable coronary syndromes and coronary stent implant. Serp-1 treatment was safe with significant reductions in troponin and creatinine kinase MB (CKMB), biomarkers of myocardial damage [24], [25]. Periodontal disease is predicted to increase inflammation and plaque growth, but anti-inflammatory treatment has not been tested for the capacity to reduce restenosis during active chronic periodontal bacterial infections. Both thrombotic and thrombolytic serine protease cascades as well as chemokines are upregulated at sites of arterial injury often initiating cellular invasion and even activation. Further it is not as yet known whether activation of serine protease coagulation pathways and/or chemokine: GAG interactions have central roles in Pg induced plaque growth after BA, nor if targeted inhibition of these selected pathways can alter plaque growth after Pg infection.

We investigate here the potential for chronic oral *P. gingivalis* infection to modify balloon angioplasty (BA)-induced plaque growth in hyperlipidemic ApoE^−/−^ mice and examine the capacity of purified anti-inflammatory viral proteins alone, M-T7 and Serp-1, without antibiotic treatment, to reduce plaque growth after BA during *P. gingivalis* infection.

## Methods

### Microbial inocula

*P. gingivalis* FDC 381 (ATCC Manassas, VA, USA) was cultured both in mycoplasma broth and blood agar plates and grown anaerobically at 37°C, as described [26], [27]. Cells were harvested and mixed equally with sterile 4% (w/v) low viscosity carboxymethylcellulose (CMC; Sigma-Aldrich, St. Louis, MO) in phosphate buffered saline (PBS) and used for oral lavage and infection (5×10^9^ bacteria per mL) [27].

### Anti-inflammatory protein source and purification

Serp-1 protein was provided by Viron Therapeutics Inc. (London, ON, Canada) and was purified from recombinant Chinese hamster ovary (CHO) cell supernatants ≥99% by sequential column chromatographic separation [21]–[25], [28]–[30] as previously described. A baculovirus expression system in *Spodoptera frugiperda*, Sf 21 (Invitrogen) and *Trichoplusia ni*, High Five (Invitrogen) cells were used, as previously described, for the expression of M-T7. In brief, the N-terminal His-tagged M-T7 protein was purified by Co-NTA (Nickel-Nitrilo-triacetic acid, Sigma) column chromatography and purity verified by SDS-PAGE, Coomassie/Silver staining and western blot analysis [19], [20], [31], [32].

### Mouse aortic angioplasty model

All animal studies were approved by the University of Florida Institutional Animal Care and Use Committee (IACUC Protocol #F173) and conform to the Guide for the Care and Use of Laboratory Animals (United States National Institutes of Health). The right iliac artery of twenty five eight week old ApoE^−/−^ mice (The Jackson Laboratory, Bar Harbor, ME, U.S.A.) under general anesthesia was exposed by laparotomy and a 0.62-mm caliber microcatheter balloon (1.5 mm×6 mm (MED PLUS perfecseal, Inc., Oshkosh, WI) inserted using a surgical microscope [23]–[26]. The balloon was inflated, advanced retrograde to the thoracic aorta and pulled back 3 times, inducing endothelial disruption and inflammation and simulating restenosis. Anti-inflammatory proteins, Serp-1 (15 µg) or M-T7 (6 µg), or control sterile saline were injected intravenously immediately post-BA.

### *P. gingivalis* infection and oral plaque sampling

**Figure 1:**
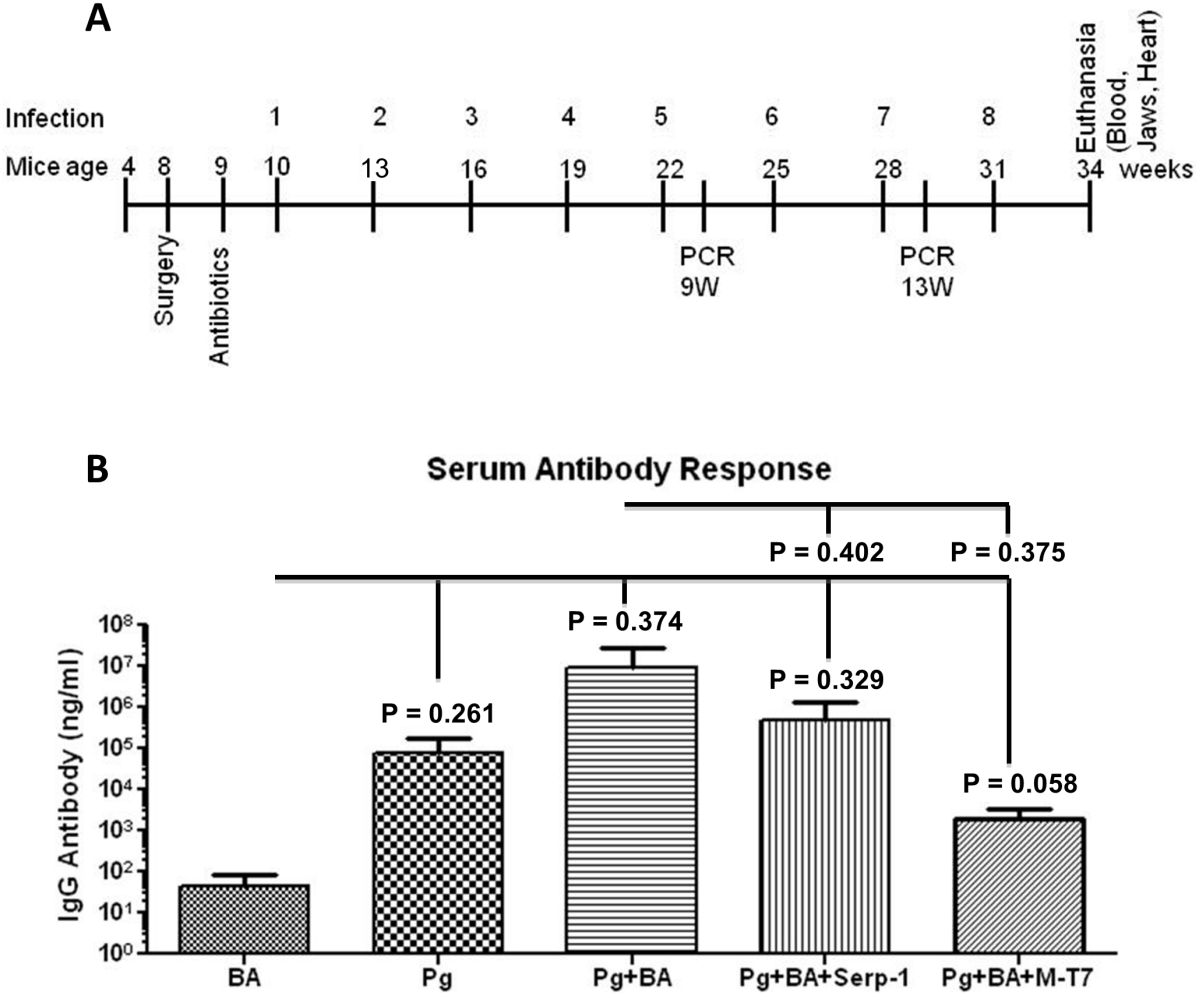
Experimental scheme and serum IgG levels in mice. (A) Schematic diagram illustrating experimental design and time course. (B) Serum *P. gingivalis* IgG antibody levels in ApoE^−/−^ mice following 24 weeks oral infection (n = 3–5). Bar graphs show the mean ± SD IgG levels in serum from mice infected with *P. gingivalis* alone, *P. gingivalis* + BA with Serp-1 or M-T7, or from BA mice (P = 0.34 and 0.37, respectively). *Pg* - *P. gingivalis*; BA - balloon angioplasty.

Monomicrobial oral infection and plaque sampling methodology are described elsewhere [26], [27], [33]. ApoE^−/−^ mice used to examine the role of oral pathogens in atherosclerotic plaque growth [5], [6], [27] were housed in microisolator cages and fed standard chow and water *ad libitum*. Mice were randomized into five groups after BA (Gr I = Sham-infected control + BA; Gr II = *P. gingivalis*; Gr III = *P. gingivalis* + BA; Gr IV =  *P. gingivalis* + BA + Serp-1; Gr V =  *P. gingivalis* + BA + M-T7) and infected as per the diagram (Figure 1A). Mice were administered kanamycin and ampicillin daily for 10 days in the drinking water and the oral cavity lavaged with 0.12% chlorhexidine gluconate (Peridex: 3 M ESPE Dental Products, St. Paul, MN) mouth rinse [27], [33] to decrease endogenous flora and to enhance *P. gingivalis* colonization [27]. 10^9^ cells in 0.2 ml per mouse were administered orally for 4 consecutive days per week, every 3 weeks (8 infection periods) to mimic chronic exposure during 24 weeks (Fig. 1A). Sham-infected mice (n = 5) received vehicle (sterile 4% CMC) only. Oral dental plaque samples from isoflurane anesthetized mice were collected post-infection as described [26], [27]. In order to monitor the oral infection with minimal disruption of biofilms, a total of 2 post-infection oral plaque samples (following the 5^th^ and 7^th^ infections) were collected from infected mice (Figure. 1A).

Following 24 weeks of infection, mice were euthanized and blood, jaws, aorta, heart, spleen, liver, and kidneys were collected for analysis. Sera were stored at −20°C for immunoglobulin G (IgG) antibody analysis [26], [27], [33]. The maxillar and mandibular regions were resected, autoclaved, and mechanically defleshed for analysis of horizontal alveolar bone loss. Maxillae were also resected and fixed in 10% buffered formalin and decalcified with Immunocal (Decal Chemical Corporation, Tallman, NY) for 28 days at 4°C for histologic analysis.

### Detection of *P. gingivalis* genomic DNA in oral plaque

Genomic DNA was isolated from mouse oral plaque using the Wizard Genomic DNA Purification Kit (Promega, Madison, WI) following manufacturer’s protocol [27], [28]. PCR was performed with a Bio-Rad thermal cycler using 16S rRNA gene species-specific oligonucleotide primers: 5’-TGTAGATGACTGATGGTGAAAACC-3’ (forward), 5’-ACGTCATCCCCACCTTCCTC-3′ (reverse). Genomic DNA extracted from *P. gingivalis* FDC 381 served as positive and PCR without template DNA served as negative controls. PCR products were separated by 1.5% agarose gel electrophoresis and bands visualized using BioRad Gel Doc XR/Chemidoc Gel Documentation System (BioRad, CA, USA).

### Serum IgG antibody analysis

Diluted mouse sera (1∶100 for IgG) were reacted with whole *P. gingivalis* coated with 0.5% formalin in buffered saline for 2 h at room temperature and subsequently goat anti-mouse IgG, conjugated to alkaline phosphatase (1∶5000) (Bethyl Laboratories, Montgomery, TX) and *p*-Nitrophenyl phosphate (Sigma-Aldrich). Reactions were stopped by 3 M NaOH and absorbance analyzed at OD_405_ using a Bio-Rad Microplate Reader. Mouse serum IgG antibody levels were calculated using a gravimetric standard curve, consisting of 8 mouse IgG concentrations (Sigma-Aldrich) coated onto microtiter plates.

### Morphometric analysis

Mouse jaws were immersed in 3% (v/v) hydrogen peroxide overnight, air dried and stained with 0.1% (w/v) aqueous methylene blue to delineate the cemento-enamel junction (CEJ) [34]. Digital images of mouse buccal and lingual root surfaces of molar teeth were captured under a 10X stereo dissecting microscope (SteReo Discovery V8; Carl Zeiss Microimaging, Inc, Thornwood, NY), after superimposition of buccal and lingual cusps to ensure reproducibility. Horizontal alveolar bone loss was measured from CEJ to alveolar bone crest (ABC) using the calibrated line tool (AxioVision LE 29A software version 4.6.3.). Means of duplicate measurements performed by two blinded individual examiners are reported.

Paraffin-embedded 5 µm cross-sections were Hematoxylin and eosin stained and the aortic plaque measured using an Olympus system with Olympus DP7 color video camera attached to an Olympus BX51 microscope with the ImagePro MC 6.0 software program standardized to microscopic objective (Olympus America, Center Valley, PA) [19]–[23], [29]–[31], [33], [35], [36]. The mean cross-sectional intimal area or intimal thickness normalized to medial thickness for each aortic section (ascending, thoracic, or abdominal aorta) or for combined data for all aortic specimens was used for statistical analyses.

### Immunohistochemistry

Aortic specimens were stained for macrophage, TLR4 and MyD88 (Myeloid differentiation marker 88) [37]. Formalin fixed tissue sections were labeled using an ABC kit (Vector Laboratories, Burlingame, USA) per manufacturer’s protocol. Blocked tissue sections were labeled using a 1∶100 dilution of specific primary antibodies [(macrophage: rat monoclonal anti-mouse CD11b to macrophage: ab56297 and secondary antibody: rat IgG (Biotin) ab6733) (TLR: rabbit polyclonal anti-mouse to TLR4: ab47093 and secondary antibody: rabbit IgG (Biotin) ab6720) (MyD88: rabbit polyclonal anti-mouse to MyD88: (HFL-296): sc-11356 (Santa Cruz Biotechnology INC., CA, U.S.A)] (Abcam Inc, Cambridge, MA) overnight followed by 1∶250 dilution of biotin-conjugated secondary antibodies [28]–[31], [34], [35]. Diaminobenzidine (Sigma-Aldrich, St. Louis, USA) was used for detection with hematoxylin counterstain. Specificity of staining was determined by omission of primary antibody or irrelevant primary antibody [29]–[31], [35], [36]. Positively stained cells were counted in three high power fields (HPF, 100X) in intimal, medial and adventitial layers of each aortic section, and mean numbers of cells calculated.

### Statistical analysis

All data is presented as mean ± SD or ± SE (Prism 4, GraphPad software, San Diego, CA or Statview software) with *P* values calculated using Kruskal Walis ANOVA with Dunn’s correction for multiple comparisons and Mann-Whitney Student *t* test [19]–[23], [26], [28]–[31], [35], [36]. *P* value less than 0.05 were considered significant.

## Results

### Detection of *P. gingivalis* after oral infection

Oral dental plaques collected post-5^th^ infection demonstrated that all the mice were positive for *P. gingivalis* in *Pg, Pg* + BA and *Pg* + BA + Serp-1 groups and 4 out of 5 mice in the *P. gingivalis* + BA + M-T7 group were positive. At post-7 weeks infection, all mice were positive for *P. gingivalis.* Neither anti-inflammatory protein treatment significantly reduced detectable *P. gingivalis* in the oral cavity. No sham-infected mice were positive for *P. gingivalis* at the two time points examined.

### Antibody response to oral *P. gingivalis* is detected during infection

When evaluated for up to 24 weeks, all mice in the *P. gingivalis* + BA group developed elevated IgG antibody to *P. gingivalis* compared to sham-infected mice with BA, but this did not reach significance (P = 0.34) (Figure 1B). The IgG antibody levels in mice treated with Serp-1 and M-T7 with BA were lower than in untreated mice with *P. gingivalis*-infection (P = 0.37) (Figure 1B). Further, IgG antibody levels in mice treated with Serp-1 were higher than with M-T7 treatment, suggesting differing immune responses after each specific treatment.

### *P. gingivalis* infection induced alveolar bone resorption

**Figure 2:**
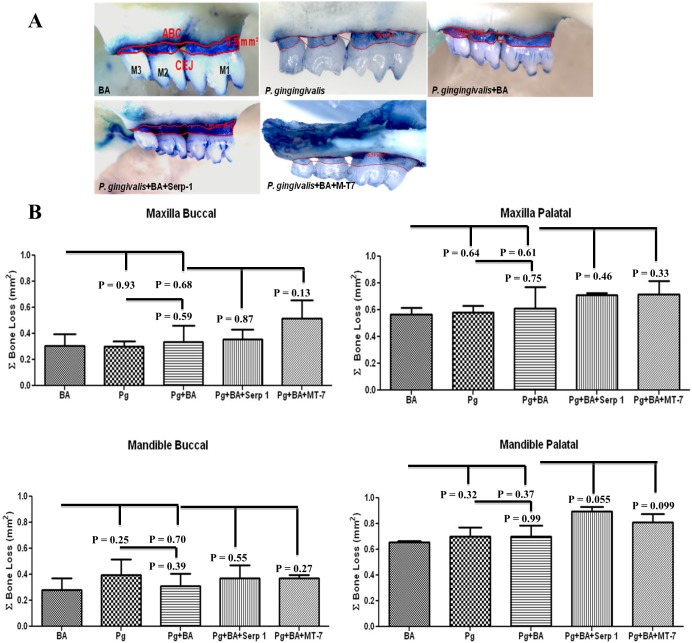
Morphometric evaluation of horizontal alveolar bone loss. (A) Representative ApoE^−/−^ mouse left maxilla with BA showing palatal horizontal bone loss area by morphometry (n = 3–5, magnification-10X). The outline represents the area of horizontal alveolar bone resorption (mm^2^). M1, M2, M3 are molars. (B) Bar graphs depicting bone loss in each of four quadrants; Maxilla (buccal and palatal) and mandible (buccal and palatal) (n = 3–5). No significant differences (P≤0.05) were observed between the treatment groups.

Progression of PD resulting from *P. gingivalis* infection was examined by measuring alveolar bone resorption (ABR) (Table 1; Figure 2A and 2B). Higher horizontal ABR was detected in both the mandible and maxilla of the palatal surface than the buccal surface of all *P. gingivalis* infected mice and sham-infected mice and BA. No observable differences in ABR were found between Serp-1 or M-T7 treated mice compared to sham infected mice (P>0.05).

**Table 1:** Morphometric evaluation of horizontal area alveolar bone resorption induced by *P. gingivalis*.

Groups/Infection/Balloon angioplasty/Serpin	Maxilla		Mandible	
	Buccal	Lingual	Buccal	Lingual
I Sham-infected + BA	0.30±0.09*	0.57±0.05	0.28±0.09	0.66±0.01
II *P. gingivalis*	0.30±0.04	0.58±0.05	0.40±0.12	0.67±0.07
III *P. gingivalis* + BA	0.34±0.12	0.61±0.16	0.31±0.09	0.70±0.08
IV *P. gingivalis* + BA + Serp-1	0.36±0.78	0.71±0.01	0.37±0.01	0.89±0.04
V *P. gingivalis* + BA + M-T7	0.52±0.14	0.72±0.10	0.37±0.02	0.81±0.06

Alveolar bone resorption (ABR) area measured between cementoenamel junction (CEJ) to the alveolar bone crest (ABC) on the buccal and palatal surfaces of the roots of all molars in mice (mean value in mm^2^± SD).

*Mean value in mm^2^ and standard deviation from 3–5 mice per group measured using AxioVision line tool software. Mice jaw images captured at 10 x magnification and measured between CEJ to the ABC on the buccal and palatal surfaces of the roots of all molars.

### Balloon angioplasty induced aortic plaque is increased with *P. gingivalis* infection

**Figure 3:**
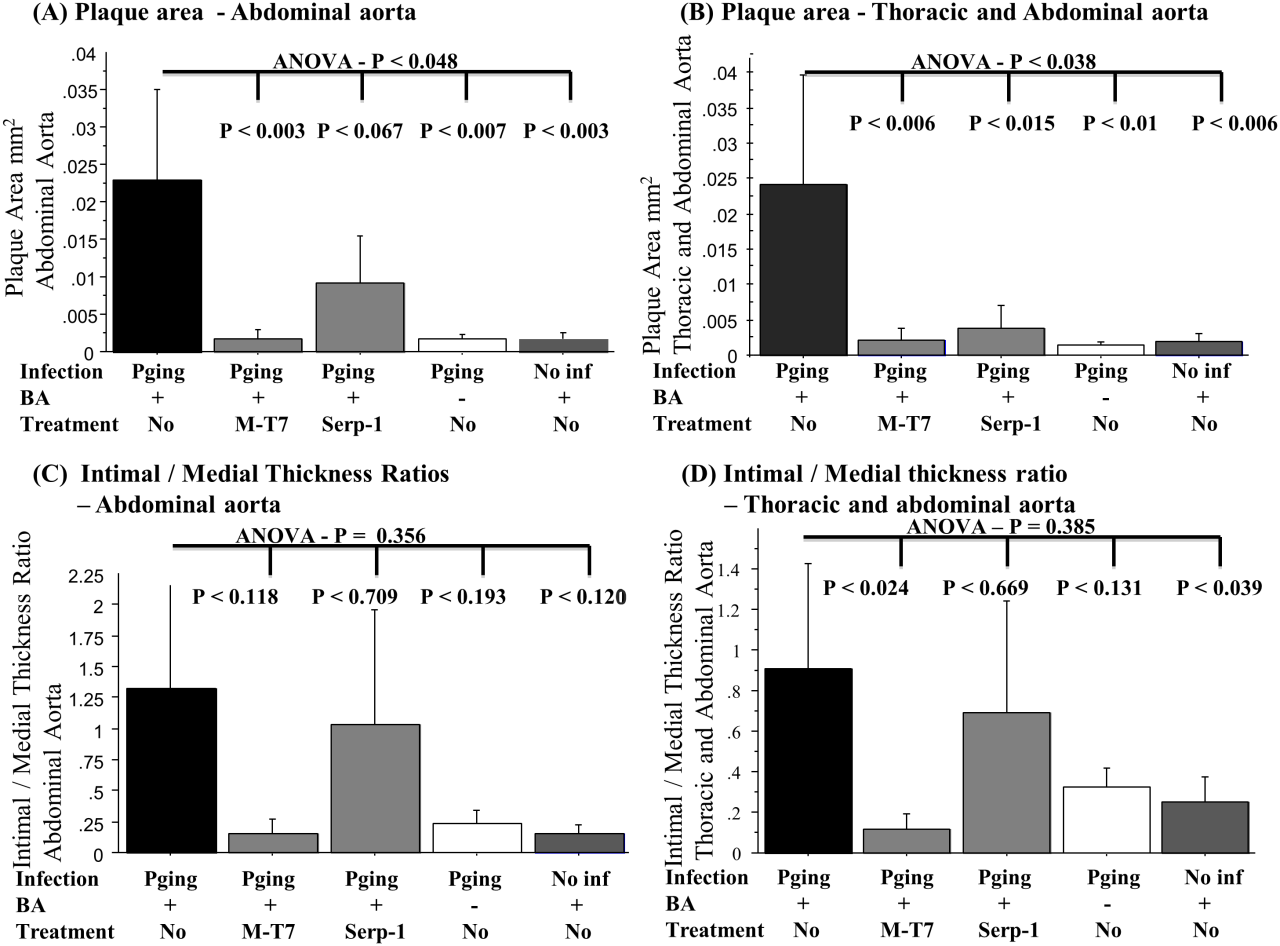
Plaque area and thickness in *P. gingivalis* infection. *P. gingivalis* infection increased plaque area significantly in ApoE^−/−^ mice after BA (A and B, P<0.006) with a trend toward increased plaque thickness (C and D, P≤0.385). Bar graphs of combined thoracic and abdominal aortic plaque area at 24 weeks (A) or abdominal aortic data alone (B) and intimal/medial thickness ratios for combined thoracic and abdominal aorta (C) or abdominal aorta alone (D) (N = 3–5 mice per group). Pg - *P. gingivalis*, Serp-1, M-T7 - anti-inflammatory protein treatments, BA - balloon angioplasty.

**Figure 4:**
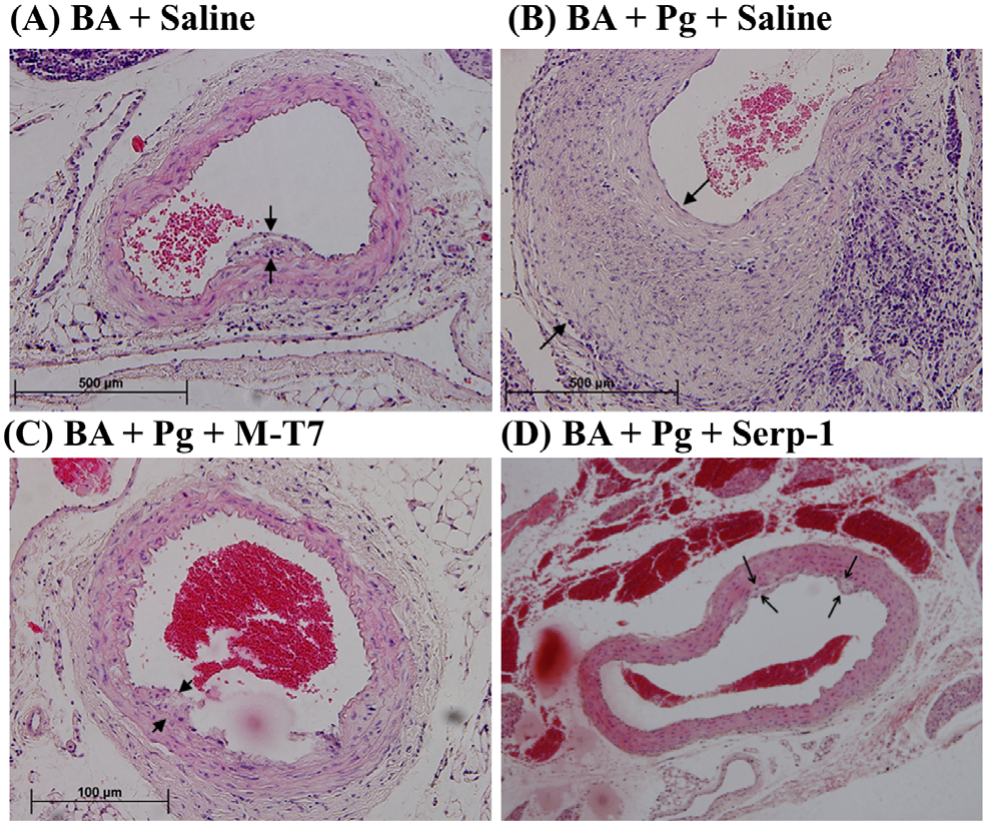
Histology of aortic plaque with *P. gingivalis* infection. Hematoxylin and eosin stained cross sections of abdominal aorta from ApoE^−/−^ mice 24 weeks after BA. *P. gingivalis* induced increase plaque thickness is significantly reduced by M-T7, with Serp-1 showing a trend towards reduction. BA with saline treatment and no *P. gingivalis* infection (A). BA with saline treatment and *P. gingivalis* infection (B). M-T7 treatment with BA and *P. gingivalis* (C). Serp-1 treatment with BA and *P. gingivalis* (D). Arrows indicate margins of intimal plaque. Arrow heads point to inflammatory mononuclear cell invasion. Magnification 200X.

Following 24 weeks of chronic oral *P. gingivalis* infection with BA, mean plaque area increased markedly throughout the aorta (*P*≤0.025) at all aortic sites from the ascending aorta to the thoracic and abdominal aortic sections, when compared to sham-infected controls with BA. Mean intimal/medial thickness ratios also increased with *P. gingivalis* infection in mice after BA (*P*≤0.039) when compared to uninfected mice. When analyzing data from the thoracic and abdominal aortic sections alone, areas of the aorta that had direct BA injury, the increase in plaque area was more pronounced as expected (Figure 3B, *P*≤0.01). Analysis of plaque area in the abdominal aorta alone, where the greatest direct BA injury occurred, demonstrated the same marked increase in plaque area in *P. gingivalis* + BA mice (Figure 3A, Figure 4A and 4B; *P*≤0.007). Analysis of intimal to medial thickness ratios, a measure of normalized thickness of aortic cross-sectional areas, did not reach significance, but demonstrated similar trends for increased plaque in the combined data for the thoracic and abdominal aorta (Figure 3D, *P* = 0.385 by ANOVA) and for the abdominal aorta data alone (Figure 3C, *P* = 0.356 by ANOVA).

### Anti-inflammatory protein treatment reduced plaque growth after balloon angioplasty during active *P. gingivalis* infection

**Figure 5:**
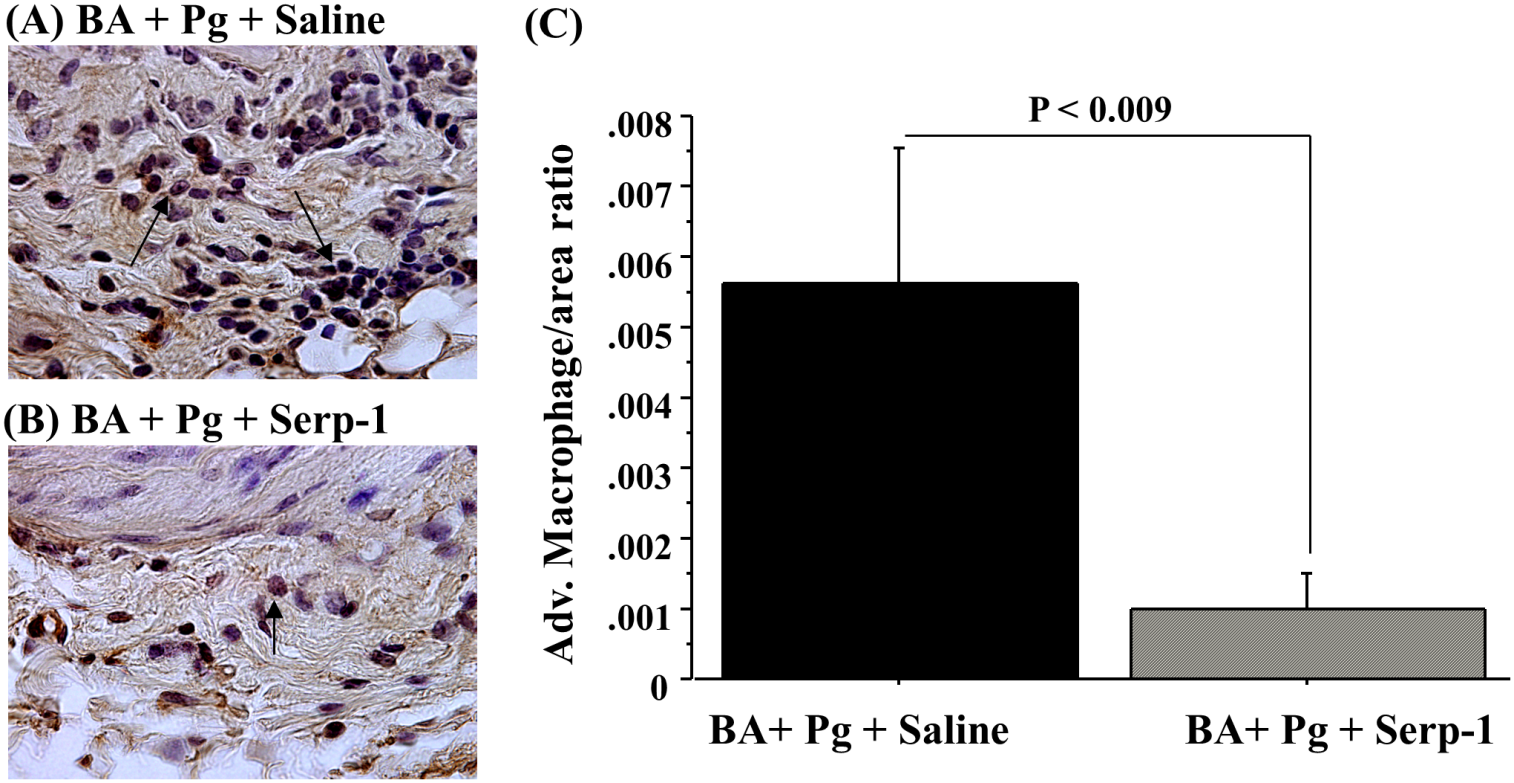
Immunohistochemistry of adventitial layer in abdominal aorta from ApoE^−/−^ mice with *P. gingivalis* infection. (A) Saline treated mouse tissue (B) demonstrates reduced macrophage invasion with Serp-1 treatment. (C) Bar graph demonstrates significantly reduced counts of positively stained macrophage in the aortic adventitia in Serp-1 treated mice with *P. gingivalis* infection + BA (C; *P*≤0.009).

In the presence of active *P. gingivalis* oral infection and BA injury, anti-inflammatory M-T7 protein treatment significantly decreased aortic plaque area when compared to saline controls in combined analysis of all aortic sections (ascending, thoracic, and abdominal areas) (*P*≤0.0227) and also in areas with direct BA injury (thoracic and abdominal aorta, *P*≤0.006) or for abdominal aorta alone (Figure 3A, Figure 3C; *P*≤0.003). In contrast, Serp-1 treatment did not significantly reduce plaque area in *P. gingivalis* infected mice when analyzing mean data from all aortic sections (*P* = 0.243), although demonstrating a trend toward reduced plaque. When analyzing only combined thoracic and abdominal aortic data, where there was direct BA injury, Serp-1 treatment did significantly reduce plaque area (Figure 3B, Figure 4D, Figure 5C; *P*≤0.015). When analyzing the abdominal aorta alone a similar, non-significant trend toward reduced plaque was detected for Serp-1 treatment (Figure 3A, *P* = 0.067). Intimal/medial thickness ratios demonstrated a significant reduction with M-T7 treatment when combined data for all aortic areas (*P*≤0.024) were analyzed, but not with Serp-1 treatment (*P* = 0.669). Analysis of combined intimal to medial ratio data from areas of BA damage in the thoracic and abdominal aorta did not reach significance, but demonstrated similar trends (Figure 3C and 3D).

### Immunohistochemical Analysis demonstrates reduced inflammatory mononuclear cell invasion after BA with anti-inflammatory protein treatment

Both M-T7 and Serp-1 (Figure 5B and 5C; *P*≤0.010; Serp-1 shown) treatments significantly decreased inflammatory macrophage infiltration in the intimal and adventitial layers of the aorta after BA during active *P. gingivalis* infection, as compared to saline control-treated mice with active *P. gingivalis* + BA (Figure 5A and 5C).

**Figure 6:**
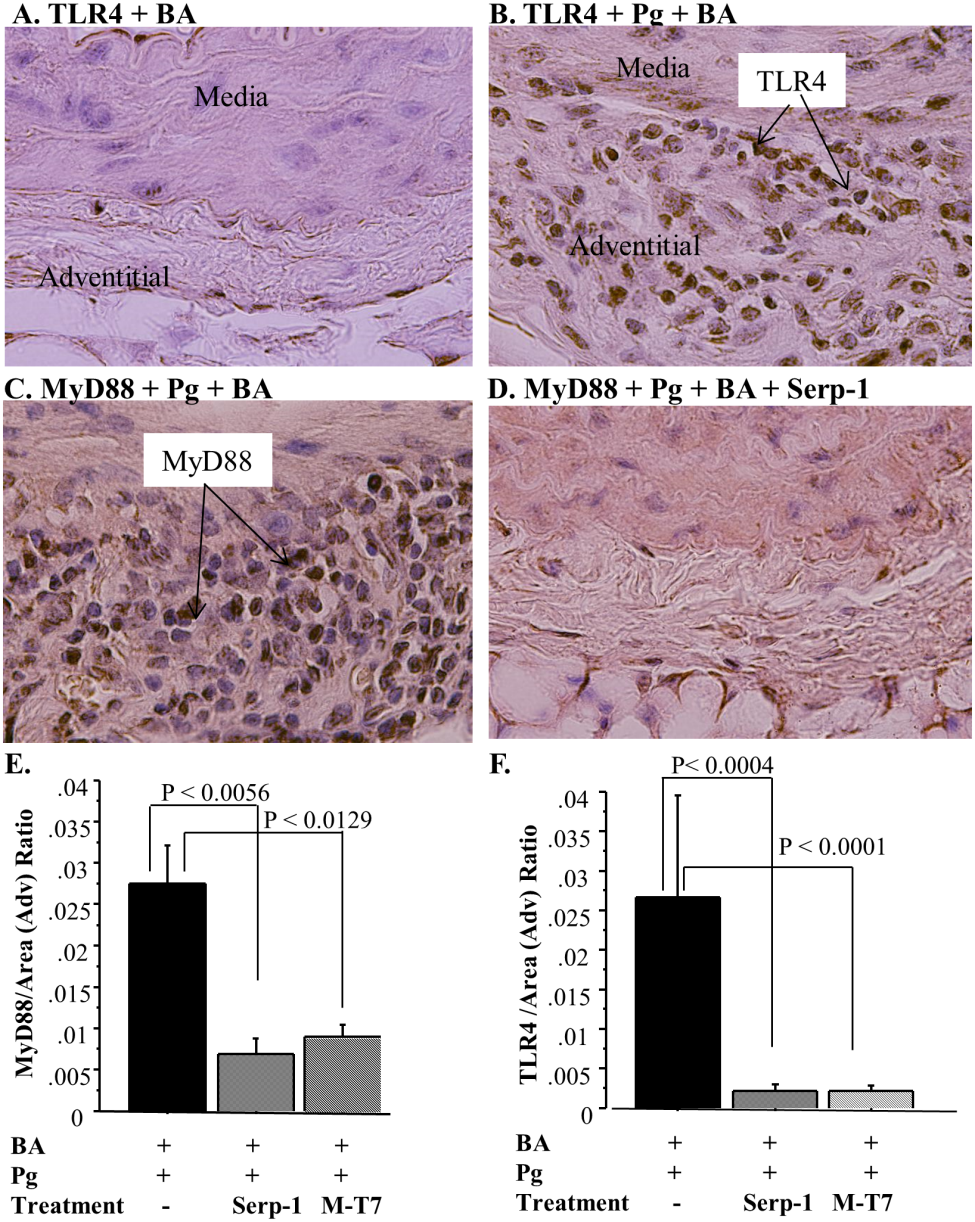
Anti-inflammatory protein treatment reduced TLR4 and MyD88 staining in mice after *P. gingivalis* infection + BA. (A) Immunohistochemical staining of TLR4 in sham-infected mice (left). (B) Increased TLR4 staining in mice infected with *P. gingivalis* + BA injury. (C) Increased MyD88 staining in mice infected with *P. gingivalis* + BA injury. (D) Decreased MyD88 staining in mice with *P. gingivalis* + BA after Serp-1 treatment. (E) Bar graph of MyD88 expression showing increased MyD88 in aortic adventitia in *P. gingivalis* infected mice + BA (24 weeks) compared to *P. gingivalis* + BA with Serp-1 (*P*≤0.056) or M-T7 treatment (*P*≤0.013). (F) Bar graph of TLR4 expression showing increased TLR4 in abdominal aortic adventitial in *P. gingivalis* + BA (24 weeks) compared to *P. gingivalis* + BA in Serp-1 (*P* = 0.0004) and in M-T7 treated mice (*P*≤0.0001).

Immunohistochemistry of the aortic adventitial layers detected increased TLR4 expression in ApoE^−/−^ mice with *P. gingivalis* infection after BA (Figure 6A and B). Treatment with M-T7 (*P*≤0.0001) or Serp-1 (*P*≤0.0004) significantly reduced TLR4 (Figure 6F). With Serp-1 treatment in *P. gingivalis* infection + BA, MyD88 expression was decreased (Figure 6D) when compared to the control, saline-treated mice with *P. gingivalis* + BA (Figure 6C). On quantitative analysis of inflammatory cells staining positively for TLR signaling adaptor protein MyD88 expression, M-T7 (*P*≤0.013) and Serp-1 (*P*≤0.006), both significantly down-regulated MyD88 (Figure 6E) in the adventitia in mice with *P. gingivalis* infection + BA when compared to saline-treated *P. gingivalis* infected mice (Figure 6).

## Discussion

Numerous epidemiological studies report associations between periodontal disease and atherosclerosis [2], [37]. Recent research has revealed profound effects of the microbiome on host immune responses and any process leading to increased inflammation at sites of BA vascular injury is postulated to increase the risk of restenosis [1]–[3]. Among known oral pathogens, *P. gingivalis* is recognized as one of the leading causative agents for periodontal disease [37], [38], a chronic inflammatory disease of the tissue, alveolar bone, and periodontal ligaments around teeth. Prior studies examined subcutaneous, *P. gingivalis* infections together with wire injury in rodent models [15], but did not assess effects of a true physiological model of chronic periodontal infection on BA-induced arterial plaque growth. Systemic infection with subcutaneous or intravenous inoculums of bacterial infection may not reproduce the effects of an ongoing oral infection on inflammation and plaque growth at remote arterial sites. The current study employed a physiological model of periodontal disease and BA to more accurately reproduce chronic and focal inflammatory states in the arterial wall. We have previously reported that Pg infection alone without BA injury can accelerate aortic plaque in hyperlipidemic ApoE^−/−^ mice [11]. Wild type C57Bl/6 mice with normal ApoE expression do not have hyperlipidemia and have minimal plaque growth. Studies are ongoing examining the effects of Pg oral infections alone on atherosclerotic plaque growth, but have not as yet been reported. Here we have focused on the rapid plaque growth seen in ApoE^null^ hyperlipidemic mice after balloon angioplasty injury with Pg infections. Further, we have used two anti-inflammatory agents that selectively block chemokine and serine protease pathways to examine the role of these two differing innate immune pathways in the accelerated plaque growth produced by BA during oral *P. gingivalis* infection.

Myxomavirus, a poxvirus lagomorph pathogen, encodes a plethora of anti-immune proteins, that target different aspects of the host immune response that are activated by viral infection, acting as a viral defense system against the host immune response to viral infections. Serp-1 is a secreted glycoprotein that inhibits the early host animal inflammatory responses to myxomavirus infection [39], [40] and is a member of the serpin superfamily inhibiting thrombotic and thrombolytic cascade proteases [40], [41]. M-T7 exhibits rabbit-species specific inhibition for interferon-γ (IFN-γ) and non-species selective chemokine-glycosaminoglycan binding in a wide range of C, CC, and CXC chemokines. Our previous work has demonstrated that M-T7 and Serp-1 inhibit BA-induced plaque growth and arterial inflammatory cell invasion after balloon injury in rodent and rabbit models [19], [31], [32].

*P. gingivalis* successfully colonized the hyperlipidemic mice upon oral infection for 24 weeks and the high IgG titer seen in infected ApoE^−/−^ mice demonstrated a specific host immune response against *P. gingivalis*. The mice also demonstrated increased alveolar bone resorption indicating development of periodontal disease and providing a model to examine infection-associated inflammation in the host artery. Markedly increased aortic plaque growth was detected after 24 weeks of infection in *P. gingivalis* infected mice with BA when compared to sham-infected controls. Treatment with viral anti-inflammatory protein M-T7 reduced plaque and inflammatory macrophage invasion after BA during *P. gingivalis* infection when compared to saline treatment, while Serp-1 also displayed similar but less effective inhibition of macrophage invasion and aortic plaque.

The reduction in recurrent plaque growth by the two anti-inflammatory proteins was not associated with a reduction in *P. gingivalis* infection suggesting that simple blockade of innate immune responses, without inhibition of bacterial proliferation can reduce plaque growth after BA during chronic oral bacterial infection. This observation is consistent with studies demonstrating reduced plaque growth in dexamethasone coated stent implants and further emphasizes the impact of the innate immune response in plaque growth after BA or with chronic *P. gingivalis* infection in periodontal disease [42], [43]. Increased expression of TLR4 and MyD88 receptors was also detected with *P. gingivalis* infection in areas of increased plaque. It has been observed that innate immune signaling occupies a prominent role in cardiovascular diseases through systemic and local effects along with attendant acquired immune responses [7]. Significant decrease in expression of TLRs by M-T7 and Serp-1 reported here supports the role of innate immune signaling in restenosis.

The greater reduction of plaque growth observed in *P. gingivalis* infected and BA injured ApoE^−/−^ mice after treatment with M-T7 treatment suggests that chemokines have a central role in early activation of the inflammatory response after angioplasty in mice with chronic *P gingivalis* oral infection. In contrast Serp-1 treatment, which inhibits cellular serine proteinases, such as plasmin or uPA, that activate matrix metalloproteinase after BA, was less effective and suggests a lesser role for the protease pathways in driving plaque growth in *P. gingivalis* infected mice after BA [28], [35]. While the chemokine–chemokine receptor interaction as well as uPA, tPA and thrombin are associated with up-regulation of inflammatory responses after arterial injury whether with BA or transplant, the relative role or impact each pathway after BA injury or in *P. gingivalis* infections has not been previously examined. We have recently reported that prolonged treatment with Serp-1 improved survival in two differing lethal viral infections in mice with associated reduction in vasculitis and arterial inflammation, further supporting the potential for use of virus-derived anti-inflammatory proteins to block arterial inflammation and plaque growth [25] The effectiveness of the anti-inflammatory compounds at reducing aortic plaque growth, in particular M-T7, even in the presence of chronic infection with a periodontal pathogen, highlights the potential therapeutic benefit of immune modulators for treatment and prevention of restenosis in patients with chronic periodontal infections. However, work with animal models with angioplasty or stent implant at sites of already developed plaque growth is necessary to fully demonstrate whether true restenosis is induced by chronic periodontal infections and/or prevented by anti-inflammatory agents.

In conclusion, chronic oral bacterial infections can accelerate plaque growth after balloon angioplasty (BA) with associated increases in inflammation. Treatment with one anti-inflammatory protein selectively targeting chemokine: GAG interactions reduced recurrent plaque growth after BA during chronic oral *P. gingivalis* infection, without associated reductions in the level of oral bacteria.
